# Polymorphisms of potential drug resistant molecular markers in *Plasmodium vivax* from China–Myanmar border during 2008‒2017

**DOI:** 10.1186/s40249-022-00964-2

**Published:** 2022-04-25

**Authors:** Zhensheng Wang, Chunyan Wei, Yunchun Pan, Zhihua Wang, Xin Ji, Qianqian Chen, Lianhui Zhang, Zenglei Wang, Heng Wang

**Affiliations:** 1 Department of Microbiology and Parasitology, Institute of Basic Medical Sciences Chinese Academy of Medical Sciences, School of Basic Medicine Peking Union Medical College, 5# Dong Dan San Tiao, Beijing, 100005 People’s Republic of China; 2 NHC Key Laboratory of Systems Biology of Pathogens, Institute of Pathogen Biology, Chinese Academy of Medical Sciences and Peking Union Medical College, 9# Dong Dan San Tiao, Beijing, People’s Republic of China; 3 No. II Department of Orthopedics, Pingdu People’s Hospital, Qingdao, Shandong 266799 People’s Republic of China; 4 Department of Immunology, Guilin Medical University, Guilin, Guangxi 541000 People’s Republic of China; 5 Institute of Chinese Materia Medica, China Academy of Chinese Medical Sciences, Beijing, People’s Republic of China

**Keywords:** *Plasmodium vivax*, Antimalarial drug, Drug resistance, Molecular marker, China–Myanmar border

## Abstract

**Background:**

*Plasmodium vivax* remains the predominant species at the China–Myanmar border, imposing a major challenge to the recent gains in regional malaria elimination. To closely supervise the emerging of drug resistance in this area, we surveyed the variations in genes potentially correlated with drug resistance in *P. vivax* parasite and the possible drug selection with time.

**Methods:**

A total of 235 *P. vivax* samples were collected from patients suffering uncomplicated malaria at Yingjiang, Tengchong, and Longling counties, and Nabang port in China, Yunnan province, and Laiza sub-township in Myanmar, from 2008 to 2017. Five potential drug resistance genes were amplified utilizing nested-PCR and analyzed, including *pvdhfr, pvdhps, pvmdr1, pvcrt-o,* and *pvk12*. The Pearson’s Chi-squared test or Fisher’s exact test were applied to determine the statistical frequency differences of mutations between categorical data.

**Results:**

The *pvdhfr* F57I/L, S58R, T61M and S117T/N presented in 40.6%, 56.7%, 40.1%, and 56.0% of the sequenced *P. vivax* isolates, and these mutations significantly decreased with years. The haplotype formed by these quadruple mutations predominated in Yingjiang, Tengchong, Longling and Nabang. While a mutation H99S/R (56.6%) dominated in Laiza and increased with time. In *pvdhps*, the A383G prevailed in 69.2% of the samples, which remained the most prevalent haplotype. However, a significant decrease of its occurrence was also noticed over the time. The S382A/C and A553G existed in 8.4% and 30.8% of the isolates, respectively. In *pvmdr1*, the mutation Y976F occurred at a low frequency in 5/232 (2.2%), while T958M was fixed and F1076L was approaching fixed (72.4%). The K10 insertion was detected at an occurrence of 33.2% in *pvcrt-o*, whereas there was no significant difference among the sites or over the time. No mutation was identified in *pvk12*.

**Conclusions:**

Mutations related with resistance to antifolate drugs are prevalent in this area, while their frequencies decrease significantly with time, suggestive of increased susceptibility of *P. vivax* parasite to antifolate drugs. Resistance to chloroquine (CQ) is possibly emerging. However, since the molecular mechanisms underneath CQ resistance is yet to be better understood, close supervision of clinical drug efficiency and continuous function investigation is urgently needed to alarm drug resistance.

**Graphical abstract:**

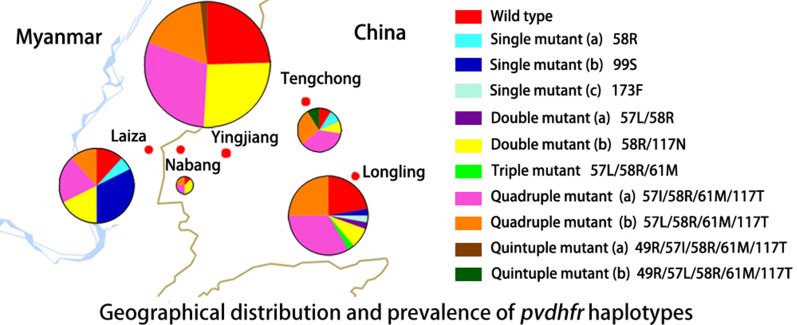

**Supplementary Information:**

The online version contains supplementary material available at 10.1186/s40249-022-00964-2.

## Background

Malaria has been a ghastly torment to human beings for millenniums and still infects 229 million people nowadays [1]. Of the five species lead to human malaria, *Plasmodium falciparum* and *P. vivax* are two major causes. Africa suffers the most morbidity and mortality resulted from *P. falciparum*, while Southeast Asia accounts for the most vivax malaria burden globally. In 2019, 51% of the world’s 6.4 million *P. vivax* cases occurred in Southeast Asia. The Greater Mekong Subregion (GMS) in Southeast Asia has targeted eliminating the menace of malaria by 2030. Of the six countries within GMS, malaria transmission levels are drastically various by country. Myanmar suffered the heaviest malaria load in GMS, whereas China reported no autochthonous malaria in 2017 and was certified indigenous malaria-free by the World Health Organization (WHO) in 2021 [2–4]. However, China still faces the challenge of maintaining the control efforts because it shares a long border with Myanmar, involving 18 counties in Yunnan Province. Thus, the cross-border transmission of malaria remains a great risk to China. *P. vivax* is the predominant malaria species in the China–Myanmar border area, which caused the recent malaria outbreaks in 2013 and 2016 [5]. Despite the cause of these outbreaks is ambiguous, increased drug resistance might be one of the factors. It is therefore an urgent priority to supervise drug resistance in *P. vivax* on the China–Myanmar border.

Increasing reports have focused on drug resistance of *P. vivax* mainly to chloroquine (CQ) and sulphadoxine-pyrimethamine (SP) regimens. CQ was first synthesized in 1934 and quickly proved to be a successful and important antimalarial agent [6]. It is now the most commonly administered drug for treating *P. vivax* infections. Other than the rapid resistance occurring in *P. falciparum* in the 1950s, CQ resistant *P. vivax* was initially reported in the late 1980s from Papua New Guinea [7] and Indonesia [8]. This was soon discovered in all around the world. High-level CQ-resistant *P. vivax* was reported in 1993 from north Myanmar [9], an area close to the southern border of China. In China, CQ has been used as the first-line drug to treat *P. vivax* along with primaquine to prevent relapse for nearly 60 years. Clinical failure or reduced efficacy of CQ-primaquine were reported in both border region [10] and the mainland [11]. SP is mainly applied in intermittent preventive treatment of malaria in pregnancy and infants, as well as in seasonal chemoprevention after its spread of resistance to *P. falciparum*. It still remains as a partner drug in artemisinin-based combination therapy (ACT) to treat sensitive *P. falciparum*. Albeit SP is not recommended to treat *P. vivax*, the common mixed-infection of *P. falciparum* and *P. vivax* in endemic area has introduced drug pressure and consequently selected SP-resistant isolates, which have been determined globally. Artemisinin resistance in *P. vivax* has not yet been reported, however, the emergence and spread of such resistance in *P. falciparum* has forewarned its appearance likely in *P. vivax*.

Molecular markers are efficient tools to monitor drug resistance. In *P. falciparum*, such markers are primarily manifested by mutant single nucleotide polymorphisms (SNPs) in related genes. CQ and SP resistance is correlated with chloroquine resistance transporter (CRT) gene and two genes encoding dihydropteroate synthase (DHPS) and dihydrofolate reductase (DHFR) enzymes, respectively, while artemisinin resistance is associated with the kelch domain of K13 gene. In addition, multidrug resistance 1 (MDR1) gene is linked to resistance to multiple antimalarials as indicated by its name. However, identification of resistant markers in *P. vivax* is particularly challenging due to the limitation of in vitro culture. Consequently, investigation of vivax resistance markers is focused on the orthologs of those identified in *P. falciparum*, mainly including *pvdhfr*, *pvdhps* [12–14], *pvmdr1*, *pvcrt-o* [15, 16], as well as *pvk12* [17–19], the ortholog of *pfk13* gene. Indeed, studies have confirmed mutations F57I/L, S58R, T61M and S117N in *pvdhfr* are responsible for pyrimethamine resistance [13, 20–22], while S382A/C, A383G, and A553G in *pvdhps* are correlated with sulfadoxine resistance [23]. Albeit polymorphisms in codons 86, 184, 1,034, 1,042 and 1,246 of the *pfmdr1* gene were reported to be associated with CQ resistance, only Y976F showed the association with decreased susceptibility to CQ, as well as to mefloquine and artemisinins [24–26]. However, the correlation of *pvcrt-o* with CQ resistance remains ambiguous. Survey of *pvcrt-o* gene failed in identifying its linkage with clinical CQ treatment failure [15], whereas analysis of mutant isoforms in yeast implies mutations in *pvcrt-o* might change the susceptibility of *P. vivax* parasite to CQ [27]. Similarly, the copy number of *pvcrt-o* was found correlated with CQ resistance in Brazilian Amazon [28], while it was not observed in samples from Papua, Indonesia [29]. Yet, the mechanism of CQ resistance still needs to be unveiled. Since the Kelch 13 (K13) gene in *P. falciparum* is considered as the main molecular marker of artemisinin resistance, likewise, its ortholog *pvk12* might be selected by drug pressure.

We report here the polymorphisms of these candidate drug resistant molecular markers, *pvdhfr*, *pvdhp*s, *pvmdr1*, *pvcrt-o* and *pvk12*, in *P. vivax* from five sites along China–Myanmar Border from 2008 to 2017 to further understand their geographical distribution and potential selection over time.

## Methods

### Sample collection

Filter paper blood samples were collected from subjects attending local clinics with fever or other symptoms suggestive of malaria, from January 2008 to December 2017 at five sites on China–Myanmar border, including Yingjiang County, Tengchong County, Longling County, Nabang Port in China, and Laiza, a sub-township in Myanmar. The diagnosis of *P. vivax* infection was carried out by microscopic examination of Giemsa-stained thin and thick blood smears. Confirmed vivax malaria cases who consented to participate provided blood spots via standard finger-prick method. The spots were dried, numbered and sealed in plastic bags and stored at −20 ℃ for later use.

### Genomic DNA extraction, amplification and sequencing

Parasite genomic DNA was extracted using the Genomic DNA Extraction Kit (NanoMagBio, Wuhan, China) via a 96-well high-throughput nucleic acid extraction instrument (NanoMagBio, Wuhan, China) in accordance with the manufacturer’s instructions. Genomic DNA was eluted in 50 μl of Tris-ethylene diamine tetraacetic acid (EDTA) buffer and stored at −20 ℃. Fragments of *pvdhfr* (755 bp), *pvdhps* (731 bp), *pvmdr1* (604 bp), *pvk12* (2,108 bp) were amplified by nested PCR in 25 μl reaction volumes as previously described [30–32], while *pvcrt-o* (1,272 bp) was amplified using regular PCR. One microliter of genomic DNA was applied as template for both nested and regular PCR in each reaction. The primers used in DNA amplification were listed in Additional file 1: Table S1. The PCR products were sequenced (Beijing TsingKe Biotech Co. Ltd., Beijing, China) commercially.

### Sequence analysis

The nucleotide and deduced amino acid sequences were assembled, aligned and compared to the reference sequences from the NCBI database using DNAMAN 9.0.1 (Lynnon Biosoft, San Ramon, CA, USA). Mutation analyses were conducted through MEGA 10.0.5 (https://www.megasoftware.net/). The accession numbers of reference sequences used in this study were as follows: *pvdhfr* (X98123), *pvdhps* (XM001617159), *pvmdr1* (AY618622), *pvcrt-o* (AF314649), and *pvk12* (PVX_083080). All sequences were submitted to GenBank (MZ960937‒MZ961394).

### Statistical analysis

The percentile data were summarized utilizing Microsoft Excel 2015 (Microsoft Corporation, San Diego, CA, USA). The Chisq.test or Fisher’s.test function in the statistical software R 3.5.3 (Lucent Technologies, Jasmine Mountain, USA) were used to assess the frequency differences of mutations between years. Pearson's chi-square test was used to determine statistical significance (*P* < 0.05). If the data did not meet the assumptions of the chi-square test (80% of the cells having expected values of five or more, and no one cell having an expected value of less than one), an adjusted chi-square test and Fisher's exact test were therefore performed.

## Results

### The origins and clinical treatment of the cases

A total of 235 *P. vivax* infected patients suffering uncomplicated malaria from January 2008 to December 2017 were enrolled in this study, with 13 (5.5%) participating during 2008‒2010, 31 (13.2%) through 2012‒2015, and a majority (191, 81.3%) between 2016 to 2017. Eighty of these samples were collected from Laiza in Myanmar, while the rest were from four sites in Yunnan province in China, including 91 from Yingjang County, 41 from Longling County, 10 from Tengchong County and 13 from Nabang Port. All patients were provided with anti-malarial therapy in accordance with the WHO guidelines for malaria treatment. Most cases (137, 58.3%) received 8-day chloroquine combined with 8-day primaquine regimen, while the rest were treated with 3-day chloroquine combined with 14-day primaquine (60, 25.5%) or a single dosage of naphthoquine phosphate (naphthoquine complexed with artemisinin, 35, 14.9%) (Table 1). DNA from their blood samples was used to amplify the *pvdhfr*, *pvdhps*, *pvmdr1*, *pvcrt-o* and *pvk12* gene sequences, and resulted in 187, 227, 232, 199, and 138 sequences.

**Table 1:** General information of the enrolled cases

General information	Case number (%)
Year	
2008‒2010	13 (5.5)
2012‒2015	31 (13.2)
2016‒2017	191 (81.3)
Gender	
Male	78 (33.2)
Female	157 (66.8)
Residence	
Laiza sub-township	80 (34.2)
Yingjiang county	91 (38.7)
Longling county	41 (17.6)
Tengchong county	10 (4.3)
Nabang port	13 (5.5)
Drug administration	
CQ (8 days) + Primaquine (8 days)	137 (58.3)
CQ (3 days) + Primaquine (14 days)	60 (25.5)
Compound naphthoquine tablet (single dosage)	35 (14. 9)
ACTs	1 (0.4)
NR	2 (0.9)

ACTs, Artemisinin-based combination therapies; CQ, chloroquine; NR, No records

### *pvdhfr* gene

Seven point mutations presented in *pvdhfr* gene, including C49R (1.1%), F57I/L (40.6%), S58R (56.7%), T61M (40.1%), H99S/R (56.6%), S117T/N (56.0%), and I173F (0.6%). Among these, the frequency of F57I/L, S58R, T61M and S117T/N decreased significantly with years, while H99S/R increased (Table 2). Haplotype analysis of *pvdhfr* revealed 11 distinct allelic forms, including the wild type (WT), haplotypes carrying a single mutation (58R, 99S, and 173F), double mutations (57L/58R and 58R/117N), triple mutations (57L/58R/61M), quadruple mutations (57I/58R/61M/117T and 57L/58R/61M/117T), and quintuple mutations (49R/57I/58R/61M/117T and 49R/57L/58R/61M/117T) (Table 3). Of note, the prevalence of haplotype containing a single mutation H99S differed significantly in samples across three time periods (0% vs 13.3% vs 33.3%, *P* = 0.006), indicating a recent selection of this mutation in this area.

**Table 2:** Prevalence of point mutations during different time periods

Mutations^a^	Number of mutations (%)			
	2008‒2010	2012‒2015	2016‒2017	Total
*pvdhfr*	*n* = 13	*n* = 30	*n* = 144	*n* = 187
C49**R**	1 (7.7)	0 (0)	1 (0.7)	2 (1.1)
F57**I/L**	11 (84.6)	18 (60.0)	47 (32.6)***	76 (40.6)
S58**R**	12 (92.3)	21 (70.0)	73 (50.7)**	106 (56.7)
T61**M**	11 (84.6)	17 (56. 7)	47 (32.6)***	75 (40.1)
H99**S/R**	2 (15.4)	10 (33.3)	91 (63.2)***	103 (56.6)
S117**T/N**	12 (92.3)	19 (63.3)	71 (49.3)**	102 (56.0)
I173**F**	0 (0)	1 (3.3)	0 (0)	1 (0.6)

*pvdhps*	*n* = 13	*n* = 30	*n* = 184	*n* = 227
A372**S**	0 (0)	0 (0)	1 (0.5)	1 (0.4)
S382**A/C**	2 (15.4)	3 (10.0)	14 (7.6)	19 (8.4)
A383**G**	12 (92.3)	28 (93.3)	117 (63.6)***	157 (69.2)
K512**E**	0 (0)	1 (3.3)	11 (6.0)	12 (5.3)
A553**G**	8 (61.5)	16 (53.3)	46 (25.0)***	70 (30.8)
E571**Q**	0 (0)	0 (0)	9 (4.9)	9 (4.0)
G626**A**	0 (0)	0 (0)	4 (2.2)	4 (1.8)
A633**S**	0 (0)	0 (0)	3 (1.6)	3 (1.3)
D639**E**	0 (0)	0 (0)	9 (4. 9)	9 (4.0)
S640**G**	0 (0)	0 (0)	9 (4. 9)	9 (4.0)
A647**S**	0 (0)	1 (3.3)	32 (17.4)	33 (15.5)

*pvmdr1*	*n* = 13	*n* = 30	*n* = 189	*n* = 232
T958**M**	13 (100)	30 (100)	189 (100)	232 (100)
Y976**F**	4 (30.8)	1 (3.3)	0 (0)	5 (2.2)
K997**R**	0 (0)	1 (3.3)	7 (3.7)	8 (3.5)
F1076**L**	8 (61.5)	17 (56.7)	143 (75.7)	168 (72.4)

*pvcrt-o*	*n* = 12	*n* = 29	*n* = 158	*n* = 199
T2**I**	0 (0)	0 (0)	1 (0.6)	1 (0.5)
**K**10 insertion	7 (58.3)	11 (37.9)	48 (30.4)	66 (33.2)
R19**C**	1 (8.3)	0 (0)	0 (0)	1 (0.5)
N57**H**	0 (0)	1 (3. 5)	0 (0)	1 (0.5)

The difference in the mutations among three periods of time was calculated by Pearson’s Chi-squared test. * *P* < 0.05, ** *P* < 0.01, *** *P* < 0.001

^a^Point mutations are shown in boldface. *Pvdhfr*, *P. vivax* dihydrofolate reductase gene; *pvdhps*, *P. vivax* dihydropteroate synthase gene; *pvmdr1*, *P. vivax* multidrug resistance 1 gene; *pvcrt-o*, *P. vivax* chloroquine resistance transporter-o gene

**Table 3:** Prevalence of *pvdhfr*, *pvdhps*, *pvmdr1*, and *pvcrt-o* haplotypes

Gene	Type	Haplotype	Number of haplotypes (%)			
			2008‒2010	2012‒2015	2016‒2017	Total
*pvdhfr*	Wild type		1 (7.7)	4 (13.3)	23 (16.0)	28 (15.0)
	Single mutant (a)	58R	0 (0)	0 (0)	2 (0.7)	2 (0.5)
	Single mutant (b)	99S	0 (0)	4 (13.3)	48 (33.3)**	52 (27.8)
	Single mutant (c)	173F	0 (0)	1 (3.3)	0 (0)	1 (0.5)
	Double mutant	57L/58R	0 (0)	1 (3.3)	0 (0)	1 (0.5)
	Double mutant	58R/117N	1 (7.7)	3 (10.0)	23 (16.0)	27 (14.4)
	Triple mutant	57L/58R/61M	0 (0)	1 (3.3)	0 (0)	1 (0.5)
	Quadruple mutant (a)	57I/58R/61M/117T	5 (38.5)	9 (30.0)	28 (19.4)	42 (22.5)
	Quadruple mutant (b)	57L/58R/61M/117T	5(38.5)	7 (23.3)	18 (12.5)*	30 (16.0)
	Quintuple mutant (a)	49R/57I/58R/61M/117T	0 (0)	0 (0)	1 (0.7)	1 (0.5)
	Quintuple mutant (b)	49R/57L/58R/61M/117T	1 (7.7)	0 (0)	0 (0)	1 (0.5)
*pvdhps*	Wild type		1 (7.7)	1 (3.3)	30 (16.3)	32 (14.1)
	Single mutant (a)	383G	4 (30.8)	7 (23.3)	63 (34.2)	74 (32.6)
	Single mutant (b)	553G	0 (0)	0 (0)	1 (0.5)	1 (0.4)
	Single mutant (c)	626A	0 (0)	3 (10.0)	1 (0.5)	4 (1.8)
	Single mutant (d)	647S	0 (0)	1 (3.3)	31 (16.8)	32 (14.1)
	Double mutant (a)	382A/383G	0 (0)	2 (6.7)	0 (0)*	2 (0.9)
	Double mutant (b)	383G/553G	6 (46.2)	14 (46. 7)	30 (16.3)***	50 (22.0)
	Double mutant (c)	512E/647S	0 (0)	0 (0)	1 (0.5)	1 (0.4)
	Double mutant (d)	626A/633S	0 (0)	0 (0)	2 (1.1)	2 (0.9)
	Triple mutant (a)	372S/626A/647S	0 (0)	0 (0)	1 (0.5)	1 (0.4)
	Triple mutant (b)	382A/383G/553G	2 (15.4)	1 (3.3)	5 (2.7)	8 (3.5)
	Triple mutant (c)	383G/512E/553G	0 (0)	1 (3.3)	1 (0.5)	2 (0.9)
	Quadruple mutant (a)	382C/383G/512E/553G	0 (0)	0 (0)	9 (4.9)	9 (4.0)
	Quadruple mutant (b)	383G/571Q/639E/640G	0 (0)	0 (0)	9 (4.9)	9 (4.0)
*pvmdr1*	Wild type		0 (0)	0 (0)	0 (0)	0 (0)
	Single mutant (a)	958 M	5 (38.5)	12 (40.0)	39 (20.6)	56 (24.1)
	Double mutant (a)	958 M/1076L	4 (30.8)	16 (53.3)	143 (75.7)***	162 (69.8)
	Double mutant (b)	958 M/997R	0 (0)	1 (3.3)	6 (3.2)	7 (3.0)
	Triple mutant (a)	958 M/976F/1076L	4 (30.8)	1 (3.3)	0 (0)	5 (2.2)
	Triple mutant (b)	958 M/997R/1076L	0 (0)	0 (0)	1 (0.5)	1 (0.4)
*pvcrt-o*	Wild type		5 (41.7)	18 (62.1)	110 (69.6)	133 (66.8)
	Single mutant (a)	K10(in)^a^	6 (50.0)	10 (34.5)	47 (29.8)	63 (31.7)
	Single mutant (b)	K10(in)/57H	0 (0)	1 (3.5)	0 (0)	1 (0.5)
	Single mutant (c)	K10(in)/19C	1 (5.1)	0 (0)	0 (0)	1 (0.5)
	Single mutant (d)	2I/K10(in)	0 (0)	0 (0)	1 (0.6)	1 (0.5)

The difference in the major haplotypes among three periods of time was calculated by corrected Pearson’s Chi-squared test or Fisher exact test. * *P* < 0.05, **P < 0.01, ***P < 0.001

^a^(in) indicates K10 insertion. *Pvdhfr*, *P. vivax* dihydrofolate reductase gene; *pvdhps*, *P. vivax* dihydropteroate synthase gene; *pvmdr1*, *P. vivax* multidrug resistance 1 gene; *pvcrt-o*, *P. vivax* chloroquine resistance transporter-o gene

**Fig. 1:**
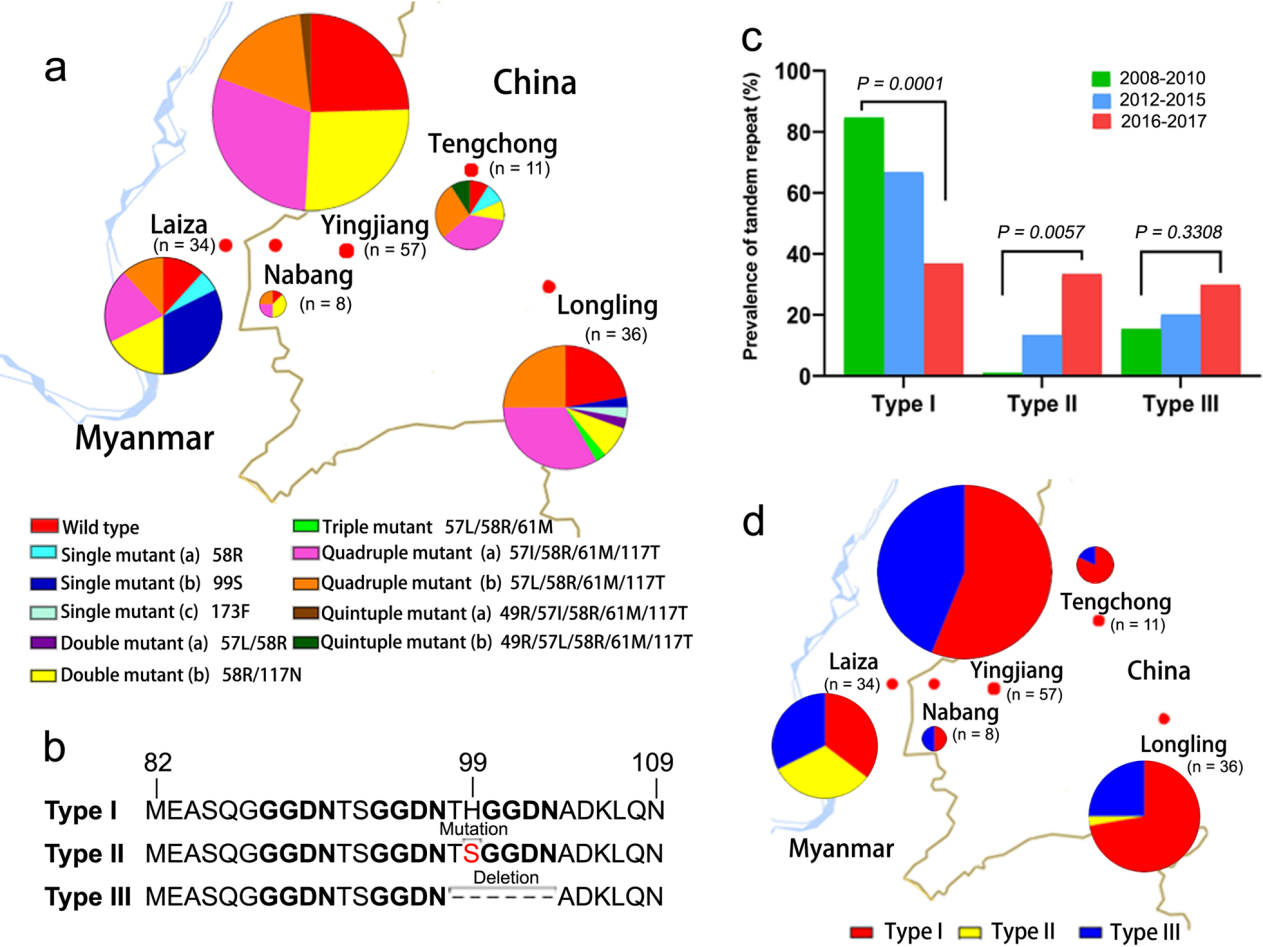
Geographical prevalence of *pvdhfr* haplotypes and tandem repeat variants. **a** The geographical distribution of *pvdhfr* haplotypes. **b** Amino-acid sequence alignment of three tandem repeat variants. Bold letters indicate the tandem repeat. **c** Frequency changes of the tandem repeat variants over time. **d** The geographical distribution of tandem repeat variants. The size of the pie represents the number of the isolates (*n*)

The geographical haplotypes prevalence analysis demonstrated that the single mutant H99S was dominant at Laiza, while it was nearly absent from other sites. However, the haplotype harboring the quadruple mutations 57I/58R/61M/117T predominated in samples from Yingjiang, Tengchong and Longling. Its related haplotype with mutations 57L/58R/61M/117T prevailed in isolates from Nabang (Fig. 1a).

Three tandem repeat variations were detected in *pvdhfr* at positions 82‒109. Type I was identical to the Sal I reference gene, type II contained a H99S mutation, and type III had a six amino-acid deletion at position 98‒103 (THGGDN) (Fig. 1b). The prevalence of type I was significantly decreased with years, and vice versa for type II. Type III tended to increase over time, though without statistically evidence (Fig. 1c). Nevertheless, type II prevailed in Laiza but nearly absent from other regions in China (Fig. 1d).

### *pvdhps* gene

**Fig. 2:**
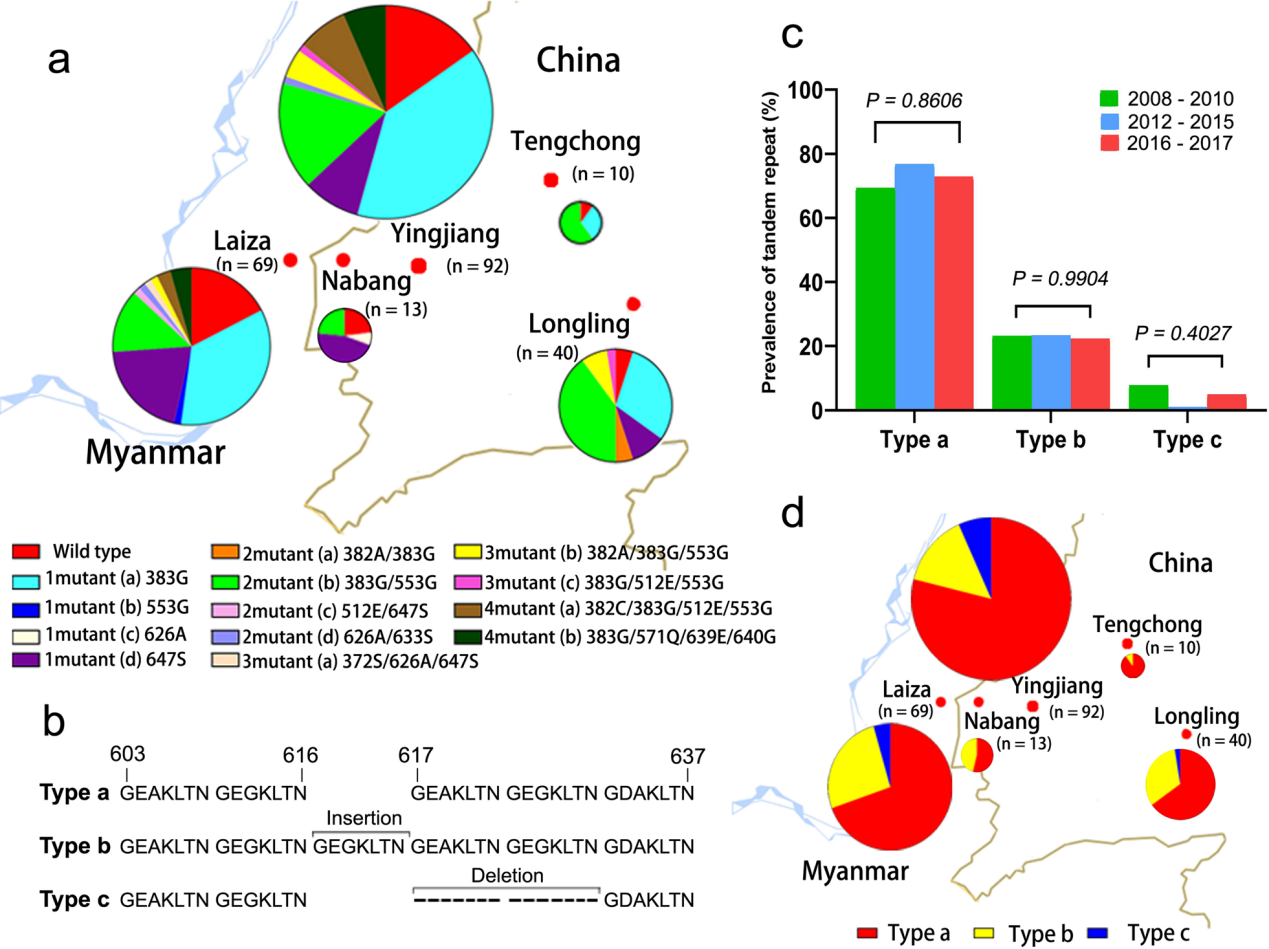
Geographical prevalence of *pvdhps* haplotypes and tandem repeat variants. **a** The geographical distribution of *pvdhps* haplotypes. **b** Amino-acid sequence alignment of three tandem repeat variants. **c** Frequency changes of tandem repeat variants over time. **d** The geographical distribution of tandem repeat variants. The size of the pie represents the number of the isolates (*n*)

Alignment of the fragments of *pvdhp*s gene discovered 11 mutations, including A372S, S382A/C, A383G, K512E, A553G, E571Q, G626A, A633S, D639E, S640G, and A647S. However, only two variants, A383G and A553G, were prevalent in the samples at an occurrence of 69.2% and 30.8%, respectively. The rest occurred at a relatively low frequency. The frequencies of A383G and A553G mutations significantly decreased with time (Table 2). This created 14 haplotypes containing WT and allelic forms harboring four single mutations (383G, 553G, 626A and 647S), four double mutations (382A/383G, 383G/553G, 512E/647S and 626A/633S), three triple mutations (372S/626A/647S, 382A/383G/553G and 383G/512E/553G), and two quadruple mutations (382C/383G/512E/553G and 383G/571Q/639E/640G). As expected, the haplotype with the single mutation A383G was the most prevalent one (32.6%), followed by A383G/A553G double mutations (22.0%) among all the *P. vivax* isolates. Interestingly, unlike the haplotype with only A383G mutation, the frequency of the haplotype with A383G/A553G significantly decreased with time (Table 3). By site, A383G remained the predominated haplotype in samples from Tengchong and Yingjiang, and A383G/A553G prevailed in Tengchong and Longling (Fig. 2a).

Three tandem repeat variant types at position 603‒637 were identified with a variable number of repeat unit ‘G (E/D) (G/A) KLTN’, much less than those reported previously in the same region [33]. Type a was identical to the *Sal*I reference sequence and carried five repeat units, type b had six units while type c held only three units (Fig. 2b). No statistical difference was found in the occurrence of these tandem repeat variations with years (Fig. 2c), however, type a remained the most common one in all sites, followed by type b (Fig. 2d).

### *pvmdr1* gene

**Fig. 3:**
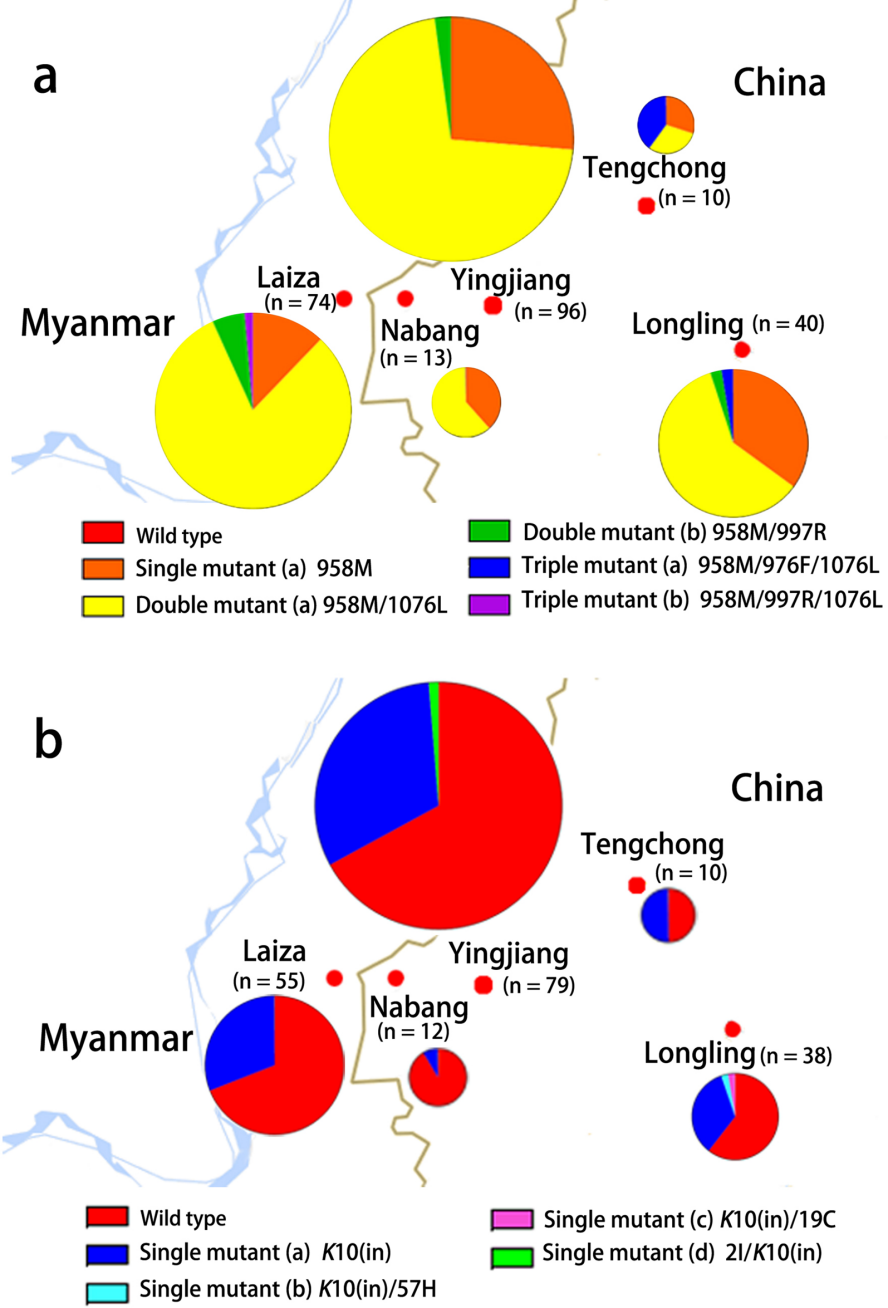
Geographical prevalence of *pvmdr1* haplotypes (**a**) and *pvcrt-o* haplotypes (**b**). The size of the pie represents the number of the isolates (*n*)

Four mutations at T958M, Y976F, K997R, and F1076L were observed in *pvmdr*1gene. The T958M mutation was fixed in all sequenced isolates, and the F1076L mutation was approaching fixed, occurring in 72.4% of the isolates. In contrast, Y976F and K997R happened in 2.2% (5/232) and 3.5% (8/232) (Table 2). This gave rise to five haplotypes comprised by a single mutation (958M), two double mutations (958M/1076L and 958M/997R), and two triple mutations (958M/976F/1076L and 958M/997R/1076L) (Table 3). It was noteworthy that the prevalence of the T958M/F1076L significantly increased over the three collection periods (30.8% vs 57.3% vs 75.7%, *P* = 0.002). Given that T958M was fixed in these samples, yet a recent selection of F1076L might have taken place in *P. vivax* in this region. Unsurprisingly, this 958M/1076L haplotype dominated in all of the sampling sites (Fig. 3a).

### *pvcrt-o* gene

Three mutations at codons 2, 19 and 57 (T2I, R19C and N57H) were detected and each occurred in one clinical isolate (Table 2). In addition, a K10 insertion was detected in 33.2% of the sequenced isolates. Thus, WT was the most prevalent haplotype (66.8%), followed by the K10 insertion (33.2%) among all sequenced *P. vivax* isolates (Fig. 3b).

### *pvk12* gene

The 2,108-bp amplified *pvk12* gene fragment contained 98.6% of the coding region. Hitherto, this gene remained conserved and no polymorphism was detected from *P. vivax* in this area.

## Discussion

China has achieved the goal set in the National Malaria Elimination Action Plan (2010‒2020) and eliminated indigenous malaria since 2017. However, border malaria remains a great concern because the risk for malaria reintroduction from the neighboring countries still exists. In particular, malaria along the China–Myanmar border area needs to be closely supervised due to the complex geographic conditions and migrant population. Antimalarial drug resistance could also jeopardize the recent gains. This study spotlighted the drug resistance in *P. vivax* at China–Myanmar border and investigated the polymorphisms in those potential drug resistance genes to better understand their epidemic pattern, geographical distribution and possible recent selection.

The spread of antifolate drug resistance in *P. vivax* parasite from GMS has been widely reported and associated with point mutations in *pvdhfr* and *pvdhps* [14, 34, 35]. As expected, results from this study demonstrated high level resistance of *P. vivax* parasite to SP at China–Myanmar border. For *pvdhfr* gene, mutations F57I/L, S58R, T61M, S117N/T and I173L/F confer resistance to pyrimethamine [36]. Among these, S58R and S117N/T occur most frequently in highly resistant *P. vivax*, particularly, S117N/T is regarded as a drug resistance determinant [21]. The quadruple mutations F57(I/L)/S58R/T61M/S117T are likely related to high level pyrimethamine resistance and clinical treatment failure [37]. Our results presented that S58R and S117N/T mutations prevailed in 56.7% and 56.0% of the isolates, similar to those reported at the same region [33], but much lower than that detected in other areas of Myanmar (71–90%) [31] and Thailand border (100%) [30]. However, the frequency of these two mutations as well as the haplotype of F57L/S58R/T61M/S117T decreased significantly with time. It is possible that the susceptibility of *P. vivax* parasite to SP has increased after its withdrawal to treat *P. falciparum* parasites which often co-infect the patients. The role of tandem repeat variation in *pvdhfr* remains ambiguous. Some reports indicate the sensitivity of *P. vivax* parasite to pyrimethamine is not related to the tandem repeats [38], because these variations occur outside of the substrate binding site [39]. Conversely, other studies report that a type of tandem repeat with four units might correlate with pyrimethamine drug resistance because of their co-existing with the quadruple 57I/58R/61M/117T mutations [40, 41]. Such type, however, was not found in this study.

Mutations S382A/C, A383G, and A553G in *pvdhps* are correlated with sulfadoxine resistance. Amongst these, mutations A383G and A553G might contribute more to the resistance, because they could cause a reduction in binding of sulfadoxine to *pvdhps* as confirmed in transgenic rodent parasites expressing *Pvdhps* protein [23, 42]. Here, we found A383G and A553G were prevailed in 69.2% and 30.8% of the isolates, respectively, similar to that reported by other studies [33]. Nevertheless, the prevalence of these two mutations significantly decreased with time, which could be another sign of increasing sensitivity of *P. vivax* parasites to SP in this region.

Recent studies monitored a decline in the therapeutic responses of *P. vivax* malaria to CQ treatment along China–Myanmar border [43, 44]. However, the molecular mechanism underlying CQ resistance in *P. vivax* remains to be fully understood. The genes *pvmdr1* and *pvcrt-o* might be involved in CQ resistance [45]. In *pvmdr1*, the in vitro sensitivity of *P. vivax* isolates carrying the Y976F shows a decrease to CQ [24, 25, 46]. This mutation is associated with CQ monotherapy failure as well [46]. The prevalence of Y976F is approaching fixation in *P. vivax* isolates from Papua Indonesian, however, here it was only detected in 2.2% of the samples, which is consistent with previous research in the same area [33]. The T958M mutation is fixed in this area, while it is confirmed irrelevant to the CQ resistance [33]. Likewise, few evidence supports the F1076L single mutation as a possible CQ resistance marker. The double mutations Y976F and F1076L are largely found from areas with CQ resistance, whereas the F1076L single mutation is more frequent in areas with rare CQ resistance [45, 47]. It is of interest that the double mutations Y976F and F1076L were found only in samples from Tengchong and Longling in our study, alarming the emerging of CQ drug resistance in this border region.

The function of *pvcrt-o* gene in CQ resistance still needs to be fully understood. The lysine (K) insertion at codon 10 might be linked with decreased CQ sensitivity [11, 24]. K10 was observed in 33.2% of the sequenced samples here, while there was no significant difference among the sites or over the time. The copy number of *pvcrt-o* might be another factor affecting CQ treatment, which was found to be correlated with CQ resistance in Brazilian Amazon [28], but this was not observed in Papua, Indonesia [29]. The expression level of *pvcrt-o* was not evaluated because dried blood spots were employed in the study. Yet, close supervision of the efficacy of CQ-primaquine regimen is important to monitor CQ resistance in this region.

Mutations in the propeller domain of *pfk13* are associated with reduced sensitivity to artemisinin and its derivatives in *P. falciparum* [48, 49]. Possibly, its orthologous gene in *P. vivax* parasite, *pvk12*, would be selected with artemisinin pressure. Hitherto, the *pvk12* gene remains conserved in this region, and only a few non-synonymous mutations were identified recently [50]. No mutation was detected in our samples, which is consistent with previous studies [19, 30]. Since CQ-primaquine remains the primary regimen to treat *P. vivax* in this area, artemisinin selection might have not yet developed.

Albeit the molecular analysis of potential drug-resistant markers could indicate the emerging and development of drug resistance in local *P. vivax* parasite population, close supervision of the clinical drug efficacy is more important to provide solid evidences and unearth firm associations between genetic variations and clinical responses.

## Conclusions

This study reported the epidemic pattern and geographical distribution of five potential drug resistant markers, *pvdhfr*, *pvdhps*, *pvmdr1*, *pvcrt-o* and *pvk12*, in *P. vivax* isolates from five sites along the China–Myanmar border during a 10-year period. Resistance to antifolate drugs is presented as expected in this area, however, the prevalence of certain mutations related to drug resistance decreased with time. Resistance to CQ is possibly emerging, therefore, close supervision of clinical drug efficiency and continuous function investigation is needed to better alarm drug resistance and direct vivax malaria treatment.

## Supplementary Information

**Additional file 1: Table S1.** PCR primer sequences for the amplification of sequences containing *P. vivax dhfr, dhps, mdr1, crt-o,* and *k12* genes.

## Data Availability

All sequences generated and analyzed during the current study were submitted to the GenBank (MZ960937‒MZ961394).

## Abbreviations

*pvdhfr*: *P. vivax* dihydrofolate reductase gene
*pvdhps*: *P. vivax* dihydropteroate synthase gene
*pvmdr1*: *P. vivax* multidrug resistance 1 gene
*pvcrt-o*: *P. vivax* chloroquine resistance transporter-o gene
*pvk12*: *P. vivax* kelch 12 gene
CQ: Chloroquine
SP: Sulphadoxine-pyrimethamine
ACT: Artemisinin-based combination therapy
WHO: World Health Organization
GMS: Greater Mekong Subregion
SNPs: Single nucleotide polymorphisms
CRT: Chloroquine resistance transporter
DHPS: Dihydropteroate synthase
DHFR: Dihydrofolate reductase
MDR1: Multidrug resistance 1
PCR: Polymerase chain reaction
