# Supplementary material for: Polymorphisms of potential drug resistant molecular markers in Plasmodium vivax from China–Myanmar border during 2008‒2017
Source: Infect Dis Poverty. 2022 Apr 25;11:43. doi: 10.1186/s40249-022-00964-2 (PMC9036727; doi:10.1186/s40249-022-00964-2)
Supplement: Supplementary file 1 — Additional file 1: Table S1. PCR primer sequences for the amplification of sequences containing P. vivax dhfr, dhps, mdr1, crt-o, and k12 genes. [file 40249_2022_964_MOESM1_ESM.docx]

Table S1. PCR primer sequences for the amplification of sequences containing *P. vivax* *dhfr, dhps, mdr1, crt-o,* and *k12* genes.

| Target gene | Round | Primer sequence 5'-3' | Product size（bp） | Tm (^o^C) | Reference |
| --- | --- | --- | --- | --- | --- |
| *pvdhfr* | 1st | F1 CACCGCACCAGTTGATTCCT | 979 | 54 | [1, 2] |
|  |  | R1 CCTCGGCGTTGTTCTTCT |  | 50 |  |
|  | 2nd | F2 CCCCACCACATAACGAAG | 755 | 50 |  |
|  |  | R2 CCCCACCTTGCTGTAAACC |  | 53 |  |
| *pvdhps* | 1st | F1 GATGGCGGTTTATTTGTCG | 1009 | 49 | [1, 2] |
|  |  | R1 GCTGATCTTTGTCTTGACG |  | 49 |  |
|  | 2nd | F2 GCTGTGGAGAGGATGTTC | 731 | 50 |  |
|  |  | R2 CCGCTCATCAGTCTGCAC |  | 53 |  |
| *Pvmdr1* | 1st | F1 ACGACATGATCCAAACGACA | 2784 | 50 | [3] |
|  |  | R1 CTTATATACGCCGTCCTGCAC |  | 54 |  |
|  | 2nd | F2 GGATAGTCATGCCCCAGGATTG | 604 | 57 | [4] |
|  |  | R2 CATCAACTTCCCGGCGTAGC |  | 56 |  |
| *pvcrt-o* | 1st | F1 AAGAGCCGTCTAGCCATCC | 1186 | 53 | [2, 4] |
|  |  | R1 AGTTTCCCTCTACACCCG |  | 50 |  |
| *pvk12* | 1st | F1 ATCCAACAGCATTTCCAACT | 2108 | 48 | [2, 5] |
|  |  | R1 CAATTAAAACGGAATGTCCA |  | 46 |  |
|  | 2nd | F2 ACCACGTGACGAGGGATAAG | 1015 | 54 |  |
|  |  | R2 AAAACGGAATGTCCAAATCG |  | 48 |  |

**Reference**

1. Ding S, Ye R, Zhang D, Sun X, Zhou H, McCutchan TF, et al. Anti-folate combination therapies and their effect on the development of drug resistance in Plasmodium vivax. Sci Rep. 2013;3:1008.

2. Tantiamornkul K, Pumpaibool T, Piriyapongsa J, Culleton R, Lek-Uthai U. The prevalence of molecular markers of drug resistance in Plasmodium vivax from the border regions of Thailand in 2008 and 2014. Int J Parasitol Drugs Drug Resist. 2018;8(2):229-237.

3. Barnadas C, Kent D, Timinao L, Iga J, Gray LR, Siba P, et al. A new high-throughput method for simultaneous detection of drug resistance associated mutations in Plasmodium vivax dhfr, dhps and mdr1 genes. Malar J. 2011;10:282.

4. Lu F, Wang B, Cao J, Sattabongkot J, Zhou H, Zhu G, et al. Prevalence of drug resistance-associated gene mutations in Plasmodium vivax in Central China. Korean J Parasitol. 2012;50(4):379-384.

5. Popovici J, Kao S, Eal L, Bin S, Kim S, Menard D. Reduced polymorphism in the Kelch propeller domain in Plasmodium vivax isolates from Cambodia. Antimicrob Agents Chemother. 2015;59(1):730-733.
